# Determinants of severe anemia among laboring mothers in Mekelle city public hospitals, Tigray region, Ethiopia

**DOI:** 10.1371/journal.pone.0186724

**Published:** 2017-11-03

**Authors:** Yirga Ebuy, Mussie Alemayehu, Mengistu Mitiku, Gelila K. Goba

**Affiliations:** 1 Department of Nutrition, Save the Children, Tigray field office, Mekelle, Ethiopia; 2 School of Public Health, College of Health Sciences, Mekelle University, Mekelle, Ethiopia; 3 Department of Obstetrics and Gynecology, University of Illinois at Chicago, Chicago, Illinois, United States of America; National Institute of Health, ITALY

## Abstract

**Introduction:**

Anemia is a global public health problem that has affected a significant number of pregnant mothers worldwide. The World Health Organization has estimated the prevalence of anemia in pregnant women at 18% and 56% in developed and developing countries, respectively. This study aimed to identify factors associated with severe anemia among laboring women in Mekelle city public hospitals, Tigray, Ethiopia.

**Methods:**

This unmatched case–control study involved 264 (88 = cases and 176 = controls) pregnant women who were recruited when they came for delivery service in Mekelle city public hospitals. The data was collected from July to August, 2016. In this study, a systematic sampling technique was used inselecting controls, but the cases were enrolled until the required sample size was reached. Bivariate and multivariate analyses were conducted to find predictors of severe anemia. Statistically significant predictors of severe anemia were identified at P-value <0.05 and 95% confidence interval.

**Results:**

A total of 264 pregnant women who came for delivery services were enrolled in this study. The major predicting variables for the occurrence of severe anemia among laboring women were residency (AOR = 3.28, 95% CI: 1.26–8.48), number of pregnancies (AOR = 2.46, 95% CI: 1.14–5.29), iron folate supplementation (AOR = 3.29, 95% CI: 1.27–8.49), dietary diversification score (AOR = 3.23, 95% CI: 1.19–8.71) and duration of menstrual cycle (AOR = 2.37, 95% CI: 1.10–5.10). The variable ‘blood loss during pregnancy’ (AOR = 6.63 95% CI: 2.96–14.86) was identified as a strong predictor of the outcome variable, severe anemia.

**Conclusion:**

This study identified determinants of severe anemia among laboring women in Mekelle city public hospitals, Northern Ethiopia. To reduce anemia, strengthening health education provision related to the importance of birth spacing and consuming diversified and iron-enriched food should be considered. Moreover, screening of pregnant women for state of anemia during their visits to health facilities, as well as de-worming for intestinal parasites infection are needed.

## Introduction

Anemia is a common disease with a varying degree of prevalence all over the world, mainly affecting pregnant mothers and children [1,2]. Anemia during pregnancy is considered severe when haemoglobin concentration is less than 7.0 g/dl, moderate when haemoglobin falls between 7.0 and 9.9 g/dl, and mild when haemoglobin concentration is from 10.0 to 11 g/dl [2,3].

In developing countries, anemia in its severe form in pregnant women remains one of the most intractable public health problems in developing countries because of various sociocultural problems including illiteracy, poverty, lack of awareness, cultural and religious taboos, poor dietary habits and high prevalence of parasitic infestation [2,4,5]. Pregnant women 15–49 years of age are among the at-risk groups. Global estimates show that more than 32.1 million pregnant women are registered to have anemia, with African and South East Asian regions accounting for 1.5% and 1.1% of pregnant women experiencing severe types of anemia [6]. In Africa only, 20.2 million women of reproductive age are found to be anaemic, of which 0.9% experience severe anaemia [6].

In Ethiopia, studies [5,7] indicate that the state of anemia varies from region to region across the nation. According to the Ethiopian Demographic and Health Survey (EDHS 2011) report, 22% of the pregnant women are anemic, with severe anemia occurring in 0.5–1.3% of them[8–9].

The extent to which anemia affects the health of mothers and their newborns is not completely established, but some studies [10,11] suggest that anemia during pregnancy has adverse effects on both the mother and the foetus. Moreover, those studies indicate that factors contributing to severe anemia among pregnant women include women’s socio-demographic, economic, nutritional and health-related status. [12,13].

Though a number of interventions such as iron and iron folate supplementations have been put in place in Ethiopia to address the high prevalence of general anemia in pregnant women, significant changes have not yet been achieved. As a result, the problem has continued. While the prevalence of anemia and its types are assessed adequately, factors associated with severe anemia have, to a large extent, remained unidentified in Ethiopia in general and the study area in particular. Identifying the main risk factors of severe anemia is fundamental for antenatal, labor and delivery caregivers to develop prevention, early detection and prompt management strategies of severe anemia during labor and delivery [14]. This study aimed to identify the determinants of severe anemia among laboring mothers who attended delivery services at Mekelle city public hospitals.

## Method and materials

The Research and Community Service ethical committee of College Health Sciences, Mekelle University, Ethiopia has approved this study. The approval number was ERC 0765/2016.

### Study area and period

The study was conducted in Mekelle city public hospitals from June 1 to August 30, 2016. Mekelle, the capital city of Tigray regional state, is 783 kilometers Addis Ababa. Mekelle has more than 300,000 inhabitants. The total number of women of reproductive age at the time of data collection was 68,093 with 10,200 of these being pregnant mothers. Mekelle city has nine governmental health centers, five general hospitals, one referral hospital and more than 74 privately-owned health facilities. The hospitals are providing curative and preventive services, with special emphasis on curative aspects. The three study hospitals (Ayder referral hospital, and Mekelle and Quiha general hospitals) are found in the Ayder, Semen and Quiha administrative districts, respectively [8].

### Study design and study population

This study was unmatched case control study that enrolled pregnant women who came for delivery services to Mekelle city hospitals. Clients with haemoglobin level of less than 7 g/dl were taken as cases, and those with normal haemoglobin level of 11–15.5 g/dl were taken as controls in the study.

### Inclusion and exclusion criteria

#### Inclusion criteria

All pregnant women who came for delivery service during the study period were included.

#### Exclusion criteria

Mothers who were severely ill and thus unable to respond to the interview were excluded.

### Sample size determination

Sample size was calculated based on the principle of two-population proportion formula by using Epi- info version 7 StatCalc assuming 95% CI, 80% power, 1:2 ratios for case and controls and odd ratio of 3. The final sample size obtained was 264, with 88 laboring women having severe anemia (<7g/dl) for cases and 176 laboring women with a normal haemoglobin level (11–15.5 g/dl) for controls.

### Sampling technique

**Fig 1:**
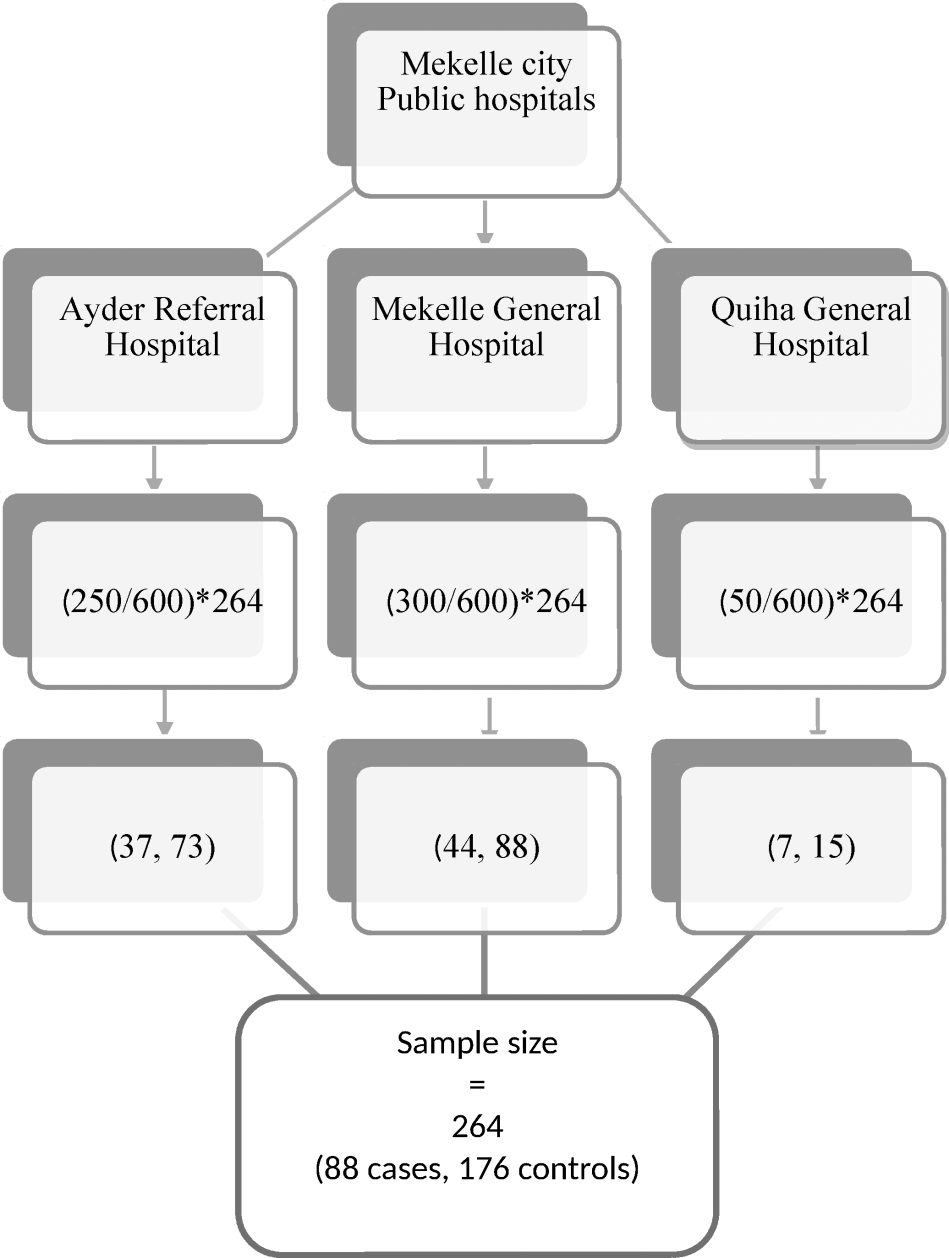
Schematic representation of sampling procedure. ↓ **=** Order of activity. * = Multiplication. / = Division. (250/600)*264 = (monthly flow of laboring mothers/Total number of laboring mothers in the three study hospitals)* total sample size. (37,73) = (number of cases, number of controls allocated to study hospital).

There are three public hospitals in Mekelle city that provide maternal health services: Ayder, Mekelle and Quiha. The three were included in the study. The number of study participants allotted to each study hospital was proportional to size allocation approach based on the previous three months laboring mothers flow. Both cases and controls were selected from the women who came for labor in the study hospitals during the study period. Systematic sampling technique was used in selection of controls, but the cases were enrolled in the study until the required sample size was reached (Fig 1).

### Study variables

The dependent variable of this study was ‘severe anemia.’ The independent variables were categorized as socio-economic and demographic characteristics, obstetric factors, maternal health conditions, and other factors including diet and history of excessive menstrual bleeding.

### Data collection tool and procedure

The laboring women were interviewed using a structured questionnaire that was adapted from other studies [2, 5, 15, 16]. The questionnaire consists of data on socio-economic and demographic characteristics, obstetric factors, maternal health conditions and other factors including diet and history of excessive menstruation bleeding and irregularity. It was administered by Bachelors of Science in Midwifery trained for this data collection In addition, three laboratory technologists were employed to measure the haemoglobin level and examine the stool samples of the laboring mothers.

### Blood specimen collection and examination

The questionnaire was administered within six hours after delivery. Blood samples were taken under strict aseptic conditions by senior laboratory technologists. The hemoglobin estimation was done using a HemoCue® B-Hemoglobin system (HemoCue AB, Ängelholm, Sweden). The HemoCue system consisted of a portable, battery-operated photometer and a supply of treated disposable cuvettes in which blood was collected. The instrument is known to be stable and durable with a sensitivity of 82.4% and specificity of 94.2% [17]. 2–3 drops of blood were drawn from the middle or ring fingers of the study participants after thoroughly cleaning using cotton soaked in 70% alcohol. After wiping away the first large drop of blood, a blood sample cuvette was immediately loaded with a large drop of blood directly from the participants’ fingers. The blood sample-loaded cuvette of the HemoCue was then immediately inserted into the device. Each hemoglobin result, expressed in gram per deciliter, was automatically displayed 30 seconds after applying blood sample in the front screen of the HemoCue machine. Results were taken from the display and documented on the questionnaire of each participant.

### Stool specimen collection and examination

Stool samples were collected from the laboring women and examined by light microscopic to identify eggs, cysts and trophozoite stages of intestinal helminthes and protozoans. Stool samples were collected using a clean and labeled container for each study participant. Stool smears were, then, prepared using physiological saline for direct microscopic detection and identification of eggs, cysts and trophozoite stages intestinal helminthes and protozoans. A portion of the stool was processed with direct microscopic technique to identify intestinal parasites immediately. A slide was prepared for each laboring woman. Direct smears were examined by three trained medical laboratory technologists microscopically, first with 10x and then with 40x objective for detection and identification of helminthes eggs, trophozoites and cysts of protozoan parasites.

### Data quality management and analysis

The questionnaire was translated into Tigrigna and back into English by translators who were not familiar with the original questionnaire. A pre-test was done on 5% of the sample at Wukro general hospital, a neighbouring hospital to the study hospitals, to check the understandability of the questionnaire. Based on the result of the pre-test, necessary corrections were made. In the laboratory, the quality of the results of the haemoglobin level determination by HemoCue and stool sample examination by microscope was ensured through appropriate blood and stool samples collections from the study participants by well-trained laboratory professionals. The validity and precision of the device (HemoCue) was checked using control samples whose hemoglobin levels were known. Data collectors were oriented about the adequacy and representativeness of the blood and stool samples that were collected from study participants.

The collected data were entered and cleaned in Epi info version 7.0. Then data were exported and analyzed using SPSS version 20. Frequencies and percentages were calculated for categorical variables. To see the association, strength and direction of the association, logistic regression was performed at a significance level of 5% and a confidence interval of 95%.

Descriptive statistics were done for variables using frequency tables, graphs, percentages and median values. Bivariate analysis was conducted to determinate variables that were associated with each independent variable. Variables found to have association with the dependent variable at P-value of 0.05 were then entered in multivariate analysis to control for the possible effect of a confounder. Variables with significant association were identified on the basis of odd ratio (OR) with 95% CI and P-values less than or equal to 0.05 to fit into the final model.

### Ethical consideration

Ethical clearance was obtained from Mekelle University College of Health Sciences School of Public Health. Moreover, letter of Cooperation was obtained from Tigray regional health bureau. Copy of the letter of cooperation was given to each study hospital. Written consent was obtained from each individual participant. All interviews with study participants were done in strict privacy.

## Result

### Socio-demographic and economic characteristics of study participants

A total of 264 pregnant women who came for delivery service were enrolled in this study, with 88 cases and 176 controls. The mean age of study participants was 28.4 with SD ± 5.8 years. Among cases, the mean age was 28.3 with SD ± 5 years; and among controls it was 28.4 with SD ± years. 29 (32.9%) of cases and 58 (32.9%) of controls were in the age range of 25–29 years. Of the 264 participants, 50(56%) of cases and 150(85%) of controls lived in urban areas. The majority of the study participants in both cases and controls were Christians; with 70 (79.5%) of cases and 153 (86.9%) of controls; and 79 (89.7%) of the cases and 171 (97.1%) of the controls being married (Table 1).

**Table 1:** Socio-demographic and economic characteristics of laboring women in Mekelle city public hospitals, 2016.

Variables	Level of Hemoglobin				
	Case (n = 88)		Control (n = 176)		Total N (%)
	No	%	No	%	
**Age of mothers**					
20–24	29	32.9	46	26.1	75(28.4)
25–29	29	32.9	58	32.9	87(33.0)
30–34	14	15.9	40	22.7	54(20.5)
35–39	16	18.1	32	18.1	48(18.1)
**Residence**					
Urban	50	56.8	150	85.2	200(75.8)
Rural	38	43.1	26	14.7	64(24.2)
**Religion**					
Christian	70	79.5	153	86.9	223(84.5)
Muslim	18	20.4	23	13	41(15.5)
**Marital status**					
Married	79	89.7	171	97.1	250(94.7)
Divorced	9	10.2	5	2.8	14(5.3)
**Mothers educational status**					
Illiterate	37	42	36	20.4	73(27.6)
Grade 1–8	23	26.1	45	25.5	68(25.8)
Grade 9–12	16	18.1	47	26.7	63(23.9)
College and above	12	13.6	48	27.2	60(22.7)
**Husband education status**					
Illiterate	23	26.1	22	12.5	45(17)
Read & write only	9	10.2	11	6.2	20(7.6)
Grade 1–8	20	22.7	30	17	50(18.9)
Grade 9–12	14	15.9	40	22.7	54(20.5)
College and above	22	25	73	41.4	95(36)
**Mother’s occupation**					
House wife	50	56.8	85	48.2	135(51.1)
Farmer	10	11.3	9	5.1	19(7.2)
Unemployed	3	1.1	7	1.7	10(3.7)
Merchant	15	13.6	35	18.1	50(18.7)
Government employ	10	11.3	41	23.2	51(19.3)
**Family monthly income (USD)**					
44–88 USD	15	41.7	28	27.2	43(33.3)
88–132 USD	9	25	32	31	41(31.8)
132–176 USD	6	16.6	17	16.5	23(17.8)
176–220 USD	6	16.6	26	25.2	22(17.1)

### Obstetric history of study participants

In this study, the predominant age category at first marriage of the study participants was ≥18 years for 51 (57.9%) of the cases and 102 (57.9%) of the controls. The mean age at first marriage was 18 years for cases and 20 years for controls. With regard to the age at first pregnancy, participants were pregnant at the age range of ≥18 years in 85 (96.5%) of cases and 171 (97.2%) of controls, with a mean age at first pregnancy of cases and controls at 20.5 and 22.6 years, respectively. Among the participants, almost two-third of cases 59 (67%) and 77 (43.7%) of controls had two and more pregnancies.

The interval between pregnancies of the study participants was more than two years in the majority of the cases and controls, 54 (81.8%) and 103 (87.2%), respectively. The majority of cases (89.7%) and controls (96.5%) attended ANC (antenatal care) during the current pregnancy, of which 41 (46.5%) of cases and 130 (73.8%) of controls attended four or more times. However, nine (10.9%) of the cases and six (3.4%) of the controls did not attend ANC. Among those who didn’t attend ANC, the reason for their absence for 11(73.3%) of them was lack of awareness and for 4(26.7%) of them, it was distance of the health facility from their kebelles. The majority of the cases, 55 (62.5%) and controls, 155 (88.1%) received iron folate supplementation during their current pregnancy. Among those mothers, 54 (96.4%) of cases and 153 (98.7%) of controls took the supplements for less than three months.

Of all the study participants, 57 (64.7%) of cases and 136 (77.2%) of controls had regular menstrual flow. Moreover, significantly higher numbers of cases (35.2%) had a prolonged menstrual period (≥5 days) than the control group with 20.4%. Cases 42 (47.7%) were more likely to have had blood loss during pregnancy than controls 21 (11.9%). Of all the cases, 21(23.8%) had had history of abortion of which 19 (90.4%) of cases experienced abortion ones. Likewise, among the 176 controls, 44(25%) had had history of abortion of which 38(86.5%) of them had had a history of at least one spontaneous abortion. Nearly three fourths (73.3%) of cases and nearly half (51%) of controls used Depo-Provera before the current pregnancy (Table 2).

**Table 2:** Obstetric history of laboring women’s in Mekelle city public hospitals, 2016.

Variable	Level of Haemoglobin		Total N (%)
	Case (n = 88)	Control (n = 176)	
**Age at first marriage**			
<18 years	37(42%)	74(42%)	111(42%)
≥18 years	51(57.9%)	102(57.9%)	153(57.9%)
**Age at first pregnancy**			
<18 years	3(3.4%)	5(2.8%)	8(3.1%)
≥18 years	85(96.5%)	171(97.2%)	256(96.9%)
**Number of deliveries**			
0	22(25%)	58(32.9%)	80(30.3%)
1–3	61(69.3%)	109(61.9%)	170(64.4%)
4–6	5(5.6%)	9(5.1%)	14(5.3%)
**Number of pregnancies**			
≤2	29(32.9%)	99(56.2%)	128(48.5%)
>2	59(67%)	77(43.7%)	138(51.5%)
**Interval between deliveries**			
≤2yrs	12(18.1%)	15(12.7%)	27 (14.6)
>2yrs	54(81.8%)	103(87.2%)	157(85.3)
**Current pregnancy ANC follow up**			
Yes	79(89.7%)	170(96.5%)	249(94.3%)
No	9(10.2%)	6(3.4%)	15(5.7%)
**Number of ANC visits**			
None	9(10.2%)	6(3.4%)	15(5.7%)
1–3 times	38(43.2%)	40(22.7%)	78(29.5%)
≥ 4 times	41(46.5%)	130(73.8%)	171(64.7%)
**First ANC visit started**			
1–3 week	27(34.1%)	91(53.5%)	118(47.4%)
4–6 week	52(65.8%)	79(46.5%)	126(51.6%)
**Iron folate supplementation**			
Yes	56(63.6%)	155(88.1%)	211(79.9)
No	32(36.3%)	21(11.9%)	53(20.1)
**Number of months iron supplemented**			
≤3	54(96.4%)	153(98.7%)	207(98.1)
>3	2(3.5%)	2(1.2%)	4(1.9)
**History of abortion**			
Yes	21(23.8%)	44(25%)	65(24.6)
No	67(76.1%)	132(75%)	199(75.4)
**Number of abortions**			
One	19(90.4%)	38(86.3%)	57(87.7)
Two	2(9.6%)	6(13.6%)	8(12.3)
**Menstruation cycle type**			
Regular	57(64.7%)	136(77.2%)	193(73.1)
Irregular	31(35.2%)	40(22.7%)	71(26.9)
**Duration of menstruation cycle**			
<5 days	57(64.7%)	140(79.5)	197(74.6)
≥5 days	31(35.2%)	36(20.4%)	67(25.4)
**Any blood loss during pregnancy**			
Yes	42(47.7%)	21(11.9%)	63(23.9)
No	46(52.2%)	155(88%)	201(76.1)
**Ever use contraceptives**			
Yes	45(51.1%)	88(50%)	133(50.4%)
No	43(48.8%)	88(50%)	131(49.6%)
**Type of contraceptives used**			
Pill	6(13.3%)	25(28.4%)	31(23.3%)
Depo-Provera	33(73.3%)	45(51.1%)	78(58.6%)
Norplant	6(13.3%)	18(20.4%)	24(18.1%)
**Possible side effects**			
Irregular period	14(31.1%)	26(29.5%)	40(30.1%)
Weight gain	3(6.6%)	10(11.3%)	13(9.7%)
No side effects	28(62.2%)	52(59%)	80(60.2%)
**History of malaria infection**			
Yes	8(9%)	11(6.2%)	19(7.2%)
No	80(90.9%)	165(93.7%)	245(92.5%)
** History of chronic illness**			
Gastritis	4(40%)	1(20%)	5(33.3%)
HIV	1(10%)	3(60%)	4(26.6%)
Hypertension	5(50%)	1(20%)	6(40.1%)
**Do you wear shoes consistently**			
Yes	86(97.7%)	173(98.2%)	259(98.1%)
No	2(2.3%)	3(1.7%)	5(1.9%)

### Dietary Intake Habit and laboratory results of study participants

Findings on the eating frequency of the laboring mothers indicated that 31 (35.2%) of cases and 24 (13.6%) of controls ate fewer than two times per day during pregnancy. Majority of the mothers had the habit of taking tea or coffee on a daily basis in 61 (69.3%) cases and 135 (76.2%) controls.

Of the 264 participants, 31 (60.7%) cases and 70 (47.6%) controls had the habit of consuming vegetables once a week during their pregnancy. Moreover, 61 (69.3%) of cases and 141 (80.1%) of controls had had the habit of eating meat and animal products, with 15 (24.5%) of cases and 51 (36.1%) of controls consuming meat and other animal products once per week.

The composite measure of dietary intake indicated that 38 (43.1%) of cases and 115 (65.3%) of controls had had low dietary diversity intake, while (20.4 and 36.5%) of cases and (24.4 and 65.3%) of controls had had moderate and high dietary diversity intake, respectively (Table 3).

**Table 3:** Dietary Intake Habit and laboratory examination of laboring women in Mekelle city public hospitals, 2016.

Variable	Level of Haemoglobin		Total N (%)
	Case (n = 88)	Control (n = 176)	
**Eating condition during pregnancy**			
Decrease	41(46.5%)	36(20.4%)	77(29.2%)
No change	29(32.9%)	74(42%)	103(39%)
Increase	18(20.4%)	66(37.5%)	84(31.8%)
**Eating frequency**			
<2 times per day	31(35.2%)	24(13.6%)	55(20.8%)
3 times per day	30(34%)	67(38%)	97(36.8%)
≥3 times per day	27(30.6%)	85(48.2%)	112(42.6%)
**Tea or coffee consumption after meal**			
Yes	61(69.3%)	135(76.7%)	203(76.8%)
No	27(30.7%)	41(23.2%)	61(23.1%)
**Frequency of tea or coffee consumption**			
Every day	50(81.9%)	111(82.2%)	167(82.2%)
Every two days	4(6.5%)	8(5.9%)	13(6.4%)
Once a week	2(3.2%)	14(10.3%)	16(7.8%)
Every two weeks	5(8.1%)	2(1.4%)	7(3.4%)
**Eat vegetables**			
Yes	51(57.9%)	147(83.5%)	200(75.7%)
No	37(42%)	29(16.4%)	64(24.2%)
**Frequency of vegetable consumption**			
Every day	1(1.9%)	14(9.5%)	15(7.5%)
Every two days	8(15.6%)	38(25.8%)	48(24%)
Once a week	31(60.7%)	70(47.6%)	101(50.5)
Every two weeks	7(13.7%)	21(14.2%)	28(14%)
Once a month	4(7.8%)	4(2.7%)	8(4)
**Eat fruits**			
Yes	49(55.6%)	156(88.6%)	207(78.4%)
No	39(44.3%)	20(11.3%)	57(21.5%)
**Frequency of fruit consumption**			
Every day	2(4%)	27(17.3%)	29(14.1)
Every two days	7(14.2%)	33(21.1%)	41(19.8%)
Once a week	23(46.9%)	57(36.5%)	81(39.1%)
Every two weeks	13(26.5%)	32(20.5%)	45(21.7%)
Once a month	4(8.1%)	7(4.4%)	11(5.3%)
**Eat meat & animal products**			
Yes	57(64.7%)	141(80.1%)	202(76.5%)
No	31(35.3%)	35(19.9%)	62(23.4%)
**Frequency of eating animal products**			
Every day	12(21%)	35(24.8%)	47(22.7%)
Every two days	3(5.2%)	18(12.7%)	21(10.1%)
Once a week	12(21%)	51(36.1%)	66(31.8%)
Every two weeks	18(31.5%)	23(16.3%)	41(19.8%)
Once a month	12(21%)	14(9.9%)	26(12.5%)
**Stool exam outcome**			
No ova parasites	38(43.1%)	117(66.4%)	155(58.7%)
Hookworm	29(32.9%)	28(15.9%)	57(21.6%)
A. lumbricoid	14(15.9%)	13(7.3%)	27(10.2%)
Giardia species	7(7.9%)	18(10.2%)	25(9.5%)

### Laboratory results of laboring women

Hookworm infection was common in the laboring women, with stool sample of 29 (32.9%) of cases and 28 (15.9%) controls containing parasitic stages.

### Predictors of severe anemia

The application of bivariate and multivariate analyses identified seven variables that independently predict severe anemia among laboring women. The variables are: residency, number of pregnancies, iron folate supplementation, duration of menstruation, blood loss during pregnancy and dietary diversification intake score.

Among the independent predictors of severe anemia among laboring women, the variable ‘blood lose during pregnancy’ has shown the strongest association (AOR = 6.63, 95% CI: 2.96–14.86) with the dependent variable followed by the variable ‘parasite infection by Ascaris lumbricoides’ (AOR = 2.94, 95% CI: 1.23–7.02). (Table 4).

**Table 4:** Determinants of severe anemia among laboring mothers in Mekelle public hospitals.

Characteristic	Level of Haemoglobin		COR (CI 95%)	AOR (CI 95%)
	Cases (<7 mg/dl)	Controls (11–15.5 mg/dl)		
**Residence**	** **	** **	** **	** **
Urban	50(56.8%)	150(85.2%)	1	1
Rural	38(43.1%)	26(14.7%)	4.39(2.42–7.93)	**3.28(1.26–8.48)**
**Gravidity**				
≤ 2	29(32.9%)	99(56.2%)	1	1
>2	59(67.0%)	77(43.7%)	2.61(1.5–4.46)	**2.46(1.14–5.29)**
**Iron folate supplementation**				
Yes	56(63.6%)	155(88.1%)	1	1
No	32(36.3%)	21(11.9%)	4.21 (2.24–7.91)	**3.29(1.27–8.49)**
**Duration of menstruation**				
<5	57(64.7%)	140(79.5)	1	1
≥5	31(35.2%)	36(20.4%)	2.11(1.19–3.74)	**2.37(1.10–5.10)**
**Dietary diversification score**				
Low	38(43.2%)	18(10.2%)	7.58(3.82–15.03)	**3.23(1.19–8.71)**
Moderate	18(20.4%)	43(24.4%)	1.50(0.76–2.95)	1.13(0.48–2.68)
High	32(36.3%)	115(65.3%)	1	1
**Mother’s educational status**				
Illiterate	37(42.0%)	36(20.4%)	1	1
Grade 1–8	23(26.1%)	45(25.5%)	0.49(0.25–0.98)	1.22(.46–3.22)
Grade 9–12	16(18.1%)	47(26.7%)	0.33(0.16–0.68)	1.81(.60–5.46)
College or above	12(13.6%)	48(27.2%)	0.24(0.11–0.53)	0.75(.23–2.47)
**Intestinal parasites infection**				
No Parasite	38(43.1%)	117(66.4%)	1	1
Hookworm	29(32.9%)	28(15.9%)	3.18(1.68–6.01)	**2.94(1.23–7.02)**
A. lumbricoid	14(15.9%)	13(7.3%)	3.31(1.43–7.67)	**4.68(1.65–13.25)**
Giardia	7(7.9%)	18(10.2%)	1.19(0.46–3.08)	1.18(0.35–4.00)
**Blood loss during pregnancy**				
Yes	42(47.7%)	21(11.9%)	6.37(3.63–12.51)	**6.63(2.96–14.86)**
No	46(52.2%)	155(88.0%)	1	1

## Discussion

The aim of this study was to identify the predicting factors of severe anemia. To this effect, data were collected from laboring women who came to Mekelle city public hospitals for delivery service. The World Health Organization estimates that Africa has the highest proportion of individuals affected by severe anemia and that in Ethiopia severe anemia is a life threatening public health problem for pregnant women.

This study found that residence of study participants, number of pregnancies (gravidity), iron folate supplementation during pregnancy, duration of menstrual cycle, intestinal parasite infection, blood loss during pregnancy and dietary diversification score were the main predictors of severe anemia among laboring mothers in Mekelle city hospitals. The seven risk factors identified in this study are discussed and recommendations for reducing risks are made in what follows.

1. Living in a rural area was positively associated with severe anemia among pregnant women who came for delivery. This is consistent with a study conducted in Bisidimo Hospital in Southeast Ethiopia [18]. In contrast to studies done in Dhaka and Gondar [13, 19], this study pointed out that residence was associated with severe anemia. The higher risk of severe anemia among women from rural areas who come to hospitals to labor may be related to a lack of information about adequate nutrition during pregnancy, economic factors, diet differences or to the inaccessibility of health care services. [20]. To reduce severe anemia, women need to be empowered economically, play a great role in decision-making, and receive counseling services for dietary diversification.
2. A statistically significant association was found between severe anemia and gravidity. A similar finding was documented in a study conducted at South West Ethiopia and Tikur Ambessa hospitals [4, 21]. This may be due the fact that multi-gravidity reduces maternal iron reserves at every pregnancy, with hemo-dilution and blood loss at each delivery. To reduce several anemia, women need to be empowered and family planning services strengthened.
3. Prolonged menstrual bleeding (≥5 days) was significantly associated with the occurrence of severe anemia among women who came for delivery. This finding is comparable concurs with a study conducted in South East Ethiopia and Welaita Sodo [18, 22]. Heavy blood loss during prolonged menstrual bleeding could lead to iron deficiency anemia. Health education of mothers might be important to counter the effects of heavy menstrual bleeding.
4. A significant association between iron folate intake and severe anemia was found, consistent with findings from other studies done among pregnant women in the Bushulo health center in Ethiopia as well as in Vietnam, Pakistan and Egypt [23–26]. Iron deficiency occurs if the amount of iron absorbed is too little to meet the body’s demand. This may be due to insufficient iron intake, reduced bioavailability of dietary iron intake or increased iron requirements during pregnancy. Declining levels of hemoglobin concentrations accompanied by the decreasing of serum levels of vitamin A, ascorbic acid, folate and B12 and multiple vitamin deficiencies have been associated with severe anemia in pregnant mothers [27]. The salutary effect of iron supplementation on improvement of haemoglobin levels in pregnancy has been documented in a study done in Dhaka city, Bangladesh [13]. The WHO recommends that all pregnant women be supplemented with 60 mg of iron daily, in a pill that also usually contains 400μg of folic acid.
5. The dietary diversification intake score was found to be an independent predictor of severe anemia among laboring mothers, those with a low dietary diversification intake score being at greater risk of developing severe anemia compared to those with a high dietary diversification intake score. This is consistent with the study done in Tigray [28]. Poor dietary diversity leads to deficiency of minerals and vitamins that may increase bioavailability of iron, thus affecting iron status. This might be due to the fact that pregnancy is a special period with increased energy and nutrient requirements, which can be fulfilled with increased meal frequency [29]. Counseling on dietary diversification and adding one extra meal during pregnancy are simple, cost-effective and sustainable methods to alleviate the problem.
6. Another statistically significant predictor of severe anemia was history of severe blood loss, with laboring mothers who had experienced severe bleeding being at greater risk of developing severe anemia compared with those who did not have prolonged bleeding [10]. This finding contradicts with the findings of a study conducted in Addis Ababa [21] where a lower risk of developing severe anaemia was obtained. This discrepancy might be due to differences in the socio-economic and education levels among study participants of Mekelle city and Addis Ababa city, as well as variation in study methods.
7. Infections with common intestinal parasites such as hookworm and Ascaris lumbricoides among laboring women were found to be independent predictors of severe anemia. This is consistent with a study conducted in South Ethiopia, Gondar and Nepal [18, 19, 30] where hookworm infection was typically found to have a significant association with severe anemia. This could be due to similar socio-economic status among residents of the study areas [23]. Hookworm control is therefore an essential component of severe anemia control. This could be achieved through community education on sanitary practices, and periodic de-worming of groups vulnerable for severe anemia, especially pregnant women.

## Supporting information

S1 File Study questionnaire. (DOCX)

S2 File Lab protocols for blood and stool sample collection. (PDF)
