# Supplementary material for: Determinants of severe anemia among laboring mothers in Mekelle city public hospitals, Tigray region, Ethiopia
Source: PLoS One. 2017 Nov 3;12(11):e0186724. doi: 10.1371/journal.pone.0186724 (PMC5669497; doi:10.1371/journal.pone.0186724)
Supplement: S1 File — (DOCX) [file pone.0186724.s001.docx]

1. **English Questionnaire**

Date Questionnaire Code No

Name of the hospital

- 1. **Consent letter**

**Introduction**

Welcome to the interview

My name is ________________________. I come from MU, College of Health Sciences to assess determinants of severe anemia in laboring women who are coming for labor and delivery service at Mekelle city public hospitals. You are selected to be one of the participants in the study and you will be kindly requested to give small amount of blood (for Hb) and stool for intestinal parasites. There will be some pain during pricking of your finger but not harmful to your health. If you agree to give samples you will be requested to answer for questionnaire including socio-demography, past and present history of pregnancy and dietary diversification after delivery within six hours. In your participation in this assessment is voluntarily and your response will remain confidential. If for any reason you wish to leave the discussion, you may do so at any time. We hope you will agree to participate as your views on the questions of this research are important. The interviews may last about 30 minutes. Do you have any question about this assessment? Are you willing to participate? Can I start the interview now?

Respondent agreed to be interviewed.

Yes, continue ‘ No, stop

Data Collector’s Name______________ Signature ________ Date_________

Result of the interview (please circle it):

1. Completed

2. Incomplete

Checked by:

Supervisor’s name_____________________ Signature ________Date_______

**1.2 Socio-economic and Demographic Information of study participants**

| **No** | **Questions** | **Alternative** | **Skip** |
| --- | --- | --- | --- |
| 101 | Age in complete years | ____________________________Years  I don’t know -------------------------------- |  |
| 102 | Residence | Urban------------------------------------------1  Rural ------------------------------------------2 |  |
| 103 | Religion | Christian --------------------------------------1  Muslim ----------------------------------------2  Other (specify)_______________ |  |
| 104 | Marital status | Single (never been married) -----------------1  Married -----------------------------------------2  Separate/divorced -----------------------------3  Other (specify)____________________ |  |
| 105 | Mother’s educational level | Illiterate -----------------------------------------1  Grade 1-8 (primary education) ---------------2  Grade 9-12 (senior secondary education)--3  College and above ----------------------------4 |  |
| 106 | Mother’s occupation | Housewife --------------------------------------1  Farmer-------------------------------------------2  Student ------------------------------------------3  Unemployed -----------------------------------4  Merchant----------------------------------------5  Daily Laborer ----------------------------------6  Government Employee ---------------------7  Other (specify) ___________________ |  |
| 107 | Husband’s educational level | Illiterate -----------------------------------------1  Read and write only ---------------------------2  Grade 1-8 (primary education) ---------------3  Grade 9-12 (senior secondary education)--4  College and above ----------------------------5 |  |
| 108 | Family’s monthly income | _______ Birr  I do not know ------------------------------ |  |
| 109 | Family’s monthly expenditure | _______ Birr  I do not know ------------------------------- |  |

**1.3. Obstetric history of study participants**

| **No** | **Questions** | **Alternative** | **Skip** |
| --- | --- | --- | --- |
| 201 | Age at first marriage | ________________________yeas |  |
| 202 | Age at first pregnancy | ______________________ years |  |
| 204 | Number of delivers? | ________________________ |  |
| 205 | How many times have you been pregnant including the current one? | _______________________ |  |
| 206 | At what interval did you deliver your babies? | ≤ 2 years---------------------------1  > 2 years---------------------------2  Not applicable--------------------3 |  |
| 207 | Do you regularly follow antenatal care in your current pregnancy? | Yes----------------------------------1  No-----------------------------------2 | If yes, go to 209 |
| 209 | How many times do you visit health facilities? | None --------------------------------1  1-3 times ---------------------------2  > 4 times or more ----------------3 |  |
| 210 | When was your first ANC visit started? | _____________________wks |  |
| 211 | Did you take iron folate during your ANC follow up? | Yes ---------------------------------1  No ----------------------------------2 |  |
| 212 | For how many months did you take iron folate? | __________________ months |  |
| 213 | Have you had abortion history? | Yes----------------------------------1  No-----------------------------------2 | If yes, go to 214 |
| 214 | If yes, how many numbers of abortions have you had before? | _________________________ |  |
| 215 | Menstrual cycle type | Regular ----------------------------1  Irregular --------------------------2 |  |
| 216 | How long does your menstruation last? | ______________________ days |  |
| 217 | Is there any blood loss in your current pregnancy? | Yes----------------------------------1  No-----------------------------------2 |  |
| 218 | Do you use contraceptive before the current pregnancy? | Yes----------------------------------1  No-----------------------------------2 | … to 219 |
| 219 | What type of contraceptive did you use? | Pills ---------------------------------1  Dipo Provera ----------------------2  Norplant ----------------------------3  IUCD -------------------------------4  Others -------------------------------5 |  |
| 220 | Have you ever developed any possible side effects related to the contraceptive? | _________________________ |  |
| 221 | Did you suffer from malaria in the past one year? | Yes----------------------------------1  No-----------------------------------2 |  |
| 222 | Was there any history of chronic diseases? | ______________________________________________________ |  |
| 223 | Do you wear shoe? | Yes ----------------------------------1  No -----------------------------------2 |  |

**1.4. Dietary Intake Habit of study participants**

| No | Questions | Alternative | Skip |
| --- | --- | --- | --- |
| 301 | Eating habit/condition during pregnancy | Decrease ---------------------------1  No change -------------------------2  Increase ----------------------------3 |  |
| 302 | How frequent do you eat your main meal per day? | <2 times ----------------------------1  3 times -----------------------------2  >3 times----------------------------3 |  |
| 303 | Do you take tea or coffee immediately after meal? | Yes-----------------------------------1  No------------------------------------2 | …to 305 |
| 304 | How frequent do you take tea or coffee immediately after meal? | Every day---------------------------1  Every two days --------------------2  Once a week------------------------3  Every two week -------------------4  Other (specify) ------------------- |  |
| 305 | Do you eat vegetables? | Yes-----------------------------------1  No------------------------------------2 | If No, go to 307 |
| 306 | How frequent do you eat vegetables? | Every day---------------------------1  Every two days --------------------2  Once a week------------------------3  Every two week -------------------4  Once a month ----------------------5  Other (specify) ------------------- |  |
| 307 | Do you eat fruit after meal? | Yes-----------------------------------1  No------------------------------------2 | …to 309 |
| 308 | How frequent do you eat fruit after meal? | Every day--------------------------1  Every two days -------------------2  Once a week-----------------------3  Every two week ------------------4  Once a month ---------------------5  Other (specify) ------------------96 |  |
| 309 | Do you eat meat and animal products? | Yes------------------------------------1  No-------------------------------------2 | …to 401 |
| 310 | How frequent do you eat meat and animal products? | Every day---------------------------1  Every two days ---------------------2  Once a week------------------------3  Every two week --------------------4  Once a month ----------------------5  Other (specify) --------------------96 |  |

**1.5 Laboratory exam**

| No | Types of laboratory test | Result | Remark |
| --- | --- | --- | --- |
| 401 | Haemoglobin | ________________________g/dl |  |
| 402 | Stool | No ova parasite-------------------------1  Hookworm------------------------------2  A.lumbricoid----------------------------3  T.trichuria-------------------------------4  Giardia-----------------------------------5  Others (specify)-----------------------6 |  |

1. **ትግርኛ ቃለ መሕትት**

ዕለት መለለይ ቑፅሪ

ናይ ሆስፒታል ስም

- 1. ናይ ፅሑፍ ፍቃዳኛነት/ሰናይ ድሌት/ መጠየቂ ቕጥዒ

ጥዕና ይሃበለይ ደሓን’ኺ/ኹም :: ነዚ ቃለ መሕትት እንኳዕ ደሓን መፃእኽን

ኣነ ይብሃል ፡፡ ዝመፃእኩሉ ካብ ዩኒቨርስቲ መቐለ ኮሌጅ ጥዕና ሳይንስ እንትኸውን ኣብ ከተማና መቐለ ዝርከቡ ናይ መንግስቲ ሆስፒታላት ግልጋሎት ወሊድ ተገልገልቲ ዝኾና ኣዴታት ሓደገኛ ናይ ደም ዋሕዲ ከምፅኡ ዝኽእሉ ምኽንያታት እንታይ ምዃኖም ንምፅናዕ እዩ ዕላማኡ ፡፡ ስለዚ ንሰን ናይዚ ፅንዓት እዚ ተሳታፊት ሓንቲ ኾይነን ብምምራፀን ብጣዕሚ ንእሹተይ ደም(ንዋሕዲ ደምን) ብተወሳኺ ድማ ሰገራ ናይ መዓንጣ ሓሳኹ ንምምርማር ብድሌተን ንክህባ ብትሕትና ንላቦ ፡፡ ካብ ኣፃብዕቲ ኢድ ደም ክውሰድ እንተሎ ንኡሽተይ ሕማም ክስመዐን ይኽእል እዩ ፡፡ ኾይኑ ግን ኣብ ጥዕነኣን ዘምፀኦ ፀገም የለን ፡፡ ናሙና ንምሃብ ፍቓደኛ ኮይነን እንድሕር ተስማዕሚዐን ኣብ ማሕበራዊን ኢኮኖሚያዊ ጉዳያት፣ምስ ጥንስን ሕርስን ኩነታት ኣመጋግባኺ ዝተተሓዙ ደም ዋሕዲ ከምትፁኡ ዝክእሉ ዝተዳለወ ቃለ መሕተት ኣብ ድሕረ ወሊድን ኣብ ውሽጢ 6፡00 ስዓታት መልሲ ክህባና ብትሕትና ንጥይቅ ፡፡ ብተወሳኺ ዝኾነ ይኹን ነዚ ቃለ መሕተት እዚ ዝወሃብ ሓበሬታ ብምስጥር ክተሓዝ ምዃኑ እዚ ቃለ መሕተት ድማ እስካብ 30 ደቒቓ ክወስድ ዘኽእል ምዃኑ ክንሕብረልኪ ንፎቱ፡፡

ምስ እዚ ተታሓሒዙ እተልዕልዮ ጥያቄ እንተሃልዩ ትጥይቂ ትክእሊ ኢኺ? በዚ መሰረት ደም ሰገራን ንምሃብ እቲ ጥያቄ ንምምላስን ፍቃደኛ ዲኺ ?

እወ ፡- ቀፅል ኣይፋሉን፡- ኣቋርፅ

መረዳእታ ኣካቢ ስም ፡- ፊርማ ዕለት

ናይ ቃለ መሕትት ውፅኢት ፡- 1. ቃለ መሕትት ብኣግባቡ ተመሊኡ

2. ብኣግባቡ ኣይተመልኣን

ዘረጋገፀ ስም ፡- ፊርማ ዕለት ____

2.2. ናይ ኣዴታት ማሕበራዊን ኢኮኖሚያዊ መረዳእታ ዝሓዘ ቃለ መሕትት

| ተ.ቑ | ሕቶታት | መማረፂ መልስታት | ናብ ጥያቄ ቁፅሪ |
| --- | --- | --- | --- |
| 101 | ናይ ኣደ ዕድመ | _______________________ ዓመት |  |
| 102 | ኣድራሻ | ከተማ ------------------------------ --1  ገጠር ------------------------------- --2 |  |
| 103 | ሃይማኖት | ክርስቲያን --------- -------------------1  ሙስልም ------------------------------2  ካልእ/ይገለፅ/ --------------------------3 |  |
| 104 | ኩነታት ሓዳር | ዘይተመርዐወት ------------------------1  ባዓልቲ ሓዳር --------------------------2  ዝተፋላለዩ/ዝተፋተሐት-----------------3  ካሊእ --------------------------------- |  |
| 105 | ናይ ኣደ ኹነታት ትምህርቲ | ዘይተምሃረት ---------------------------1  ምንባብን ምፅሓፍን ትኽእል -------------2  ካብ 1^ይ^ - 8^ይ^ ክፍሊ - - - - - - ---------3  ካብ 9^ይ^ -12^ተ^ ክፍሊ - - -- - - - - -------4  ኮሌጅ (ዩኒቨርስቲ ዝወደኣት) - - - -------5 |  |
| 106 | ናይ ባዓል ገዛኺ ኹነታት ት/ቲ | ዘይተምሃረ - - - - -- - - - - - -----------1  ምንባብን ምፅሓፍን ዝኽእል ---------- ----2  ካብ 1^ይ^ - 8^ይ^ ክፍሊ - - - - - - -----------3  ካብ 9^ይ^ -12^ተ^ ክፍሊ - - -- - - - - --------4  ኮሌጅ (ዩኒቨርስቲ ዝወደኣ) - - ------ -----5 |  |
| 107 | ናይ ኣደ ኩነታት ስራሕ | የቤት እምቤት - - - - - ------------------1  ገባር - - - - - - - - - - ------------------2  ተምሃሪት - - - - - - - -------------------3  ስራሕ ዘይብላ - - - - - ------------------4  ነጋዲት - - - - - - - - - -----------------5  መዕልታዊ ሰራሕተኛ - - -----------------6  መንግስቲ ሰራሕተኛ - - -------- ---------7  ካልእ - - - - - - - |  |
| 108 | ናይ ቤተሰብኹም ወርሓዊ ኣታዊ መጠን ክንደይ ይኸውን ? | _________________________ ብር |  |
| 109 | ናይ ቤተሰብኹም ወርሓዊ ወፃኢ መጠን ክንደይ ይኸውን ? | _________________________ ብር |  |

2.3. ምስ ጥንሲን ሕርስን ካሊኦት ተዛመዲቲ ኩነታት ዝሓዘ ቃለ መሕትት

| ተ.ቑ | ሕቶታት | መማረፂ መልሲታት | ናብ ጥያቄ ቁፅሪ |
| --- | --- | --- | --- |
| 201 | ኣብ ክንደይ ዓመቲኪ/ክን ተመርዕውኪ/ክን/? | _________________ ዓመት |  |
| 202 | ናይ መጀመርያ ጥንሲኺ/ኽን ኣብ ክንደይ ዓመት እኺ/እኺን ጠንሲኺ/ኽን ? | _________________ ዓመት |  |
| 204 | ክሳብ ሐዚ ክንደይ ግዜ ወሊድኪ/ክን ? | ___________________ ግዜ |  |
| 205 | ምስ ናይ ሐዚ ጥንሲኺ ሓዊሱ እስካብ ሐዚ ክንደይ ግዜ ጠንሲኺ ኣኺ ? | __________________ ግዜ |  |
| 206 | ደቂኺ/ውላዲኺ/ በቢ ክንደይ ግዜ ኣረሓሕቂኺ ትወሊዲዮም? | ትሕቲ 2^ተ^ ዓመት - - - - ------------1  ካብ 2^ተ^ ዓመት ንላዕሊ - - - ----------2  ኣይምልከታን - - - - - - - ----------3 |  |
| 207 | ኣብዚ ናይ ሕዚ ጥንሲኺ ናይ ቅድመ ወሊድ ምርመራ ትገብሪ ዶ ነይርኪ ? | እወ - - - - - -----------------------1  ኣይፋሉን - - -----------------------2 | ------ ናብ 209 |
| 209 | “እወ” እንተኾይኑ መልሰን እስካብ ሐዚ ክንደይ ግዜ ከይደን ኣለዋ ? | ምንም ግዜ ----------------------------1  1-3 ግዜ ---------------------------2  ኣርባዕተ ግዜ ካብኡ ንላዕሊን --------3 |  |
| 210 | መዓዝ ኢኽን/ኺ ጥንሲ ምርመራ ጀምርኪን? | _____________________ ወርሒ |  |
| 211 | ኣብ ጥንሲ እዋን ኣይረን ክኒና ወስዲኪ/ክን ዶ ነይርኪ/ክን ? | እወ --------------------------------1  ኣይፋሉን ---------------------------2 | ------ናብ 212 |
| 212 | ንክንደይ ኣዋርሕ ዝኣክል ወሲድኪ/ክን? | ____________________ወርሒ |  |
| 213 | ምንፃል ጥንሲ ኣጋጢምኪ ይፈልጥ ዶ ? | እወ -------------------------------1  ኣይፋሉን --------------------------2 | ------ ናብ 214 |
| 214 | “እወ” እንተኾይኑ ክንደይ ግዜ ይኸውን ? | ______________________ግዜ |  |
| 215 | ኩነታት ወርሓዊ ፅጊያት | ስሩዕ ------------------------------1  ዘይስሩዕ ---------------------------2 |  |
| 216 | ወርሓዊ ፅጊያት ንክንደይ ማዓልቲ ይፀንሕ? | ____________________ መዓልቲ |  |
| 217 | ኣብ ናይ ሐዚ ጥንሲኺ ናይ ደም ምፍሳስ ፀገም ኣጋጢምኪ ዶ ነይሩ ? | እወ ---------------------------------1  ኣይፋሉን ----------------------------2 |  |
| 218 | ቅድሚ ምጥናሲኺ ናይ መከላኸሊጥንሲ ትጥቀሚ ዶ ነይርኪ | እወ ---------------------------------1  ኣይፋሉን ----------------------------2 | ------ናብ 219 |
| 219 | “እወ” እንተኾይኑ እንታይ ዓይነት መከላኸሊ ትጥቀሚ ነይርኪ | ብኣፍ ዝውሓጥ ክኒን------------------1  መርፍእ/ ዲፖ/ ----------------------2  ኖርፕላንት --------------------------3  IUCD ------------------------------4  ካሊእ--------------------------------5 |  |
| 220 | ምስ መከላኸሊ ጥንሲ ዝተታሓዘ እንታይ ዓይነት ሳዕቤናት ኣጋጢምኪ ነይሩ? | ________________________________________________________ |  |
| 221 | ኣብ ዝሓለፈ ሓደ ዓመት ብሕማም ብዓሶ ሓሚምኪ ዶ ነይርኪ ? | እወ ---------------------------------1  ኣይፋሉን ----------------------------2 |  |
| 222 | ዝፍለጥ ዝኾነ ዓይነት ሕዱር ሕማም ኣለኪ ዶ? | _______________________________________________________ |  |
| 223 | ጫማ ትጥቀሚ’ዶ? | እወ ---------------------------------1  ኣይፋሉን ---------------------------2 |  |

2.4. ናይ ኣዴታት ኣመጋግባ ስርዓት ዝሓዘ ቃለ መሕትት

| ተ.ቑ | ሕቶታት | መማረፂ መልሲታት | ናብ ጥያቄ ቁፅሪ |
| --- | --- | --- | --- |
| 301 | ኩነታት አመጋግባኪ አብ ግዘ ጥንሲ እንታይ ይመስልሕ? | ይቅንስ --------------------------------1  ለውጢ የብሉን -------------------------2  ይውስክ -------------------------------3 |  |
| 302 | ኣብ ማዓልቲ ክንደይ ግዜ ትምገቢ ? | ትሕቲ ክልተ ግዜ ----------------------1  ሰለስተ ግዜ ----------------------------2  ካብ ሰለስተ ንላዕሊ ---------------------3 |  |
| 303 | ድሕሪ ምግቢ ምብላዕኺ ሽዑ ንሽዑ ሻሂ ወይ ቡና ኩሉ ግዜ ትጥቀሚ ዶ ? | እወ ------------------------------ ----1  ኣይፋሉን -----------------------------2 | ---------ናብ 305 |
| 304 | ሻሂ ወይ ቡና ምስ ምግቢ ማዓዝ ማዓዝ ትጥቀሚ ? | በቢ ማዓልቱ - - - - - - - --------------1  በቢ ክልተ ማዓልቲ - - - - --------------2  በቢ ሰሙን- - - - - - - - --------------3  በቢ ክልተ ሰሙን - - - - --------------4  ብወርሒ ሓደ ግዜ ---- - - -------------5  ካሊእ --------------------------------96 |  |
| 305 | ኣሕምልቲ ኩሉ ግዜ ዶ ትምገቢ ? | እወ -----------------------------------1  ኣይፋሉን ------------------------------2 | ---------ናብ 307 |
| 306 | ኣሕምልቲ ማዓዝ ማዓዝ ትምገቢ ? | በቢ ማዓልቱ - - - - - ------------------1  በቢ ክልተ ማዓልቲ - -------------------2  በቢ ሰሙን- - - - - - - -----------------3  በቢ ክልተ ሰሙን - - - -----------------4  ብወርሒ ሓደ ግዜ ------- ---------------5  ካሊእ -------------------------------- |  |
| 307 | ፍራምረ ድሕሪ ምግቢ ትምገቢ ዶ ? | እወ -----------------------------------1  ኣይፋሉን ------------------------------2 | ---------ናብ 309 |
| 308 | ፍራምረ ኣብ ክንደይ ማዓልታት ትወስዲ ? | በቢ ማዓልቱ - - - - - - ---------------- 1  በቢ ክልተ ማዓልቲ - - - ---------------2  በቢ ሰሙን- - - - - -- - - --------------3  በቢ ክልተ ሰሙን - - - - ------------ ---4  ብወርሒ ሓደ ግዜ - - - - ----------- ----5  ካሊእ ----------------------------- |  |
| 309 | ስጋን ካሊኦት ውፅኢት እንሰሳትን ኩሉ ግዜ ዶ ትምገቢ | እወ -----------------------------------1  ኣይፋሉን ------------------------------2 | ---------ናብ 401 |
| 310 | ስጋን ካሊኦት ውፅኢት እንሰሳትን በቢ ክንደይ ግዜ ኣከታትልኪ ትምገቢ ? | በቢ ማዓልቱ - - - - - ---------------- --1  በቢ ክልተ ማዓልቲ - -- -----------------2  በቢ ሰሙን- - - - - - ------------------3  በቢ ክልተ ሰሙን - - - ------------------4  ብወርሒ ሓደ ግዜ -----------------------5  ካሊእ ---------------------------------- |  |

**2.5. ናይ ላብራቶሪ ውፅኢት**

| ተ.ቑ | ሕቶታት | መማረፂ መልሲታት | ናብ ጥያቄ ቁፅሪ |
| --- | --- | --- | --- |
| 401 | ሄሞግሎቢን | _________________________ግ/ዲሊ |  |
| 402 | ናይ ሰገራ ምርመራ ውፅኢት | ምንም የለን ----------------------------1  ሁክዎርም -----------------------------2  ኣስካርያስ------------------------------3  ትሪኩርስ-ትርኩርያ --------------------4  ጃርዲያ -------------------------------5  ካሊእ/ይገለፅ/ ------------------------- |  |

**ንዝሃብክኒ ሓበሬታ ብጣእሚ እየ ዘመስግን የቀንየለይ!!!**
