# Supplementary material for: Determinants of severe anemia among laboring mothers in Mekelle city public hospitals, Tigray region, Ethiopia
Source: PLoS One. 2017 Nov 3;12(11):e0186724. doi: 10.1371/journal.pone.0186724 (PMC5669497; doi:10.1371/journal.pone.0186724)
Supplement: S2 File — (PDF) [file pone.0186724.s002.pdf]

STOOL EXAMINATION AND HEMOGLOBIN MEASUREMENT LABORATORY PROTOCOL

DIRECT EXAMINATION OF FAECES FOR PARASITES

Routinely faecal specimens are examined in district laboratories by direct technique. This involves:

- Reporting the appearance of the specimen and identifying any parasitic worms or tapeworm segments.
- Examining the specimen microscopically for:
  - motile parasites such as the larvae of *S. stercoralis* and trophozoites of *E. histolytica*, *G. lamblia*, and more rarely, *B. coli*,
  - helminth eggs,
  - cysts and oocysts of intestinal protozoa.

Microscopical examination of faecal specimens
Examine immediately those specimens containing blood and mucus and those that are unformed because these may contain motile trophozoites of *E. histolytica* or *G. lamblia.*

Examination of dysenteric and unformed specimens

1 Using a wire loop or piece of stick, place a small amount of specimen, to include blood and mucus on one end of a slide. Without adding saline, cover with a cover glass and using a tissue, press gently on the cover glass to make a thin preparation.

2 Place a drop of eosin reagent on the other end of the slide. Mix a small amount of the specimen with the eosin and cover with a cover glass.

3 Examine immediately the preparations microscopically, first using the 10_ objective with the condenser iris *closed sufficiently* to give good contrast. Use the 40_ objective to identify motile trophozoites, e.g. *E. histolytica* amoebae or *G. lamblia* flagellates.

Examination of semi-formed and formed faeces
1 Place a drop of fresh physiological saline on one end of a slide and a drop of iodine on the other end.

2 Using a wire loop or piece of stick mix a small amount of specimen, about 2 mg, (matchstick head amount) with the saline and a similar amount with the iodine. Make smooth *thin* preparations. Cover each preparation with a cover glass.
Important: Sample from different areas in and on the specimen or preferably mix the faeces before sampling to distribute evenly any parasites in the specimen. Do not use too much specimen otherwise the preparations will be too thick, making it difficult to detect and identify parasites.

3 Examine systematically the entire saline preparation for larvae, ciliates, helminth eggs, cysts, and oocysts. Use the 10_ objective with the condenser iris *closed sufficiently* to give good contrast.
Use the 40_ objective to assist in the detection and identification of eggs, cysts, and oocysts. Always examine several microscope fields with this objective before reporting ‘No parasites found’.

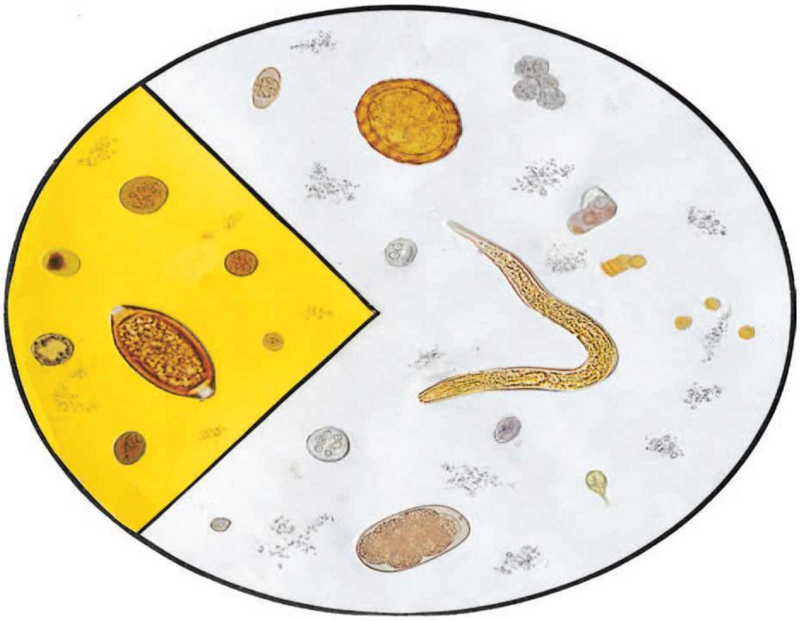

Plate 5.2 Relative sizes of trophozoites and cysts of intestinal protozoa, common nematode eggs and larva of *Strongyloides* as seen in microscope field using the 40_ objective (with 10_ eyepieces).

4 Use the iodine preparation to assist in the identification of cysts.
5 Report the number of larvae and each species of egg found in the entire saline preparation as follows:
Scanty . . . . 1–3 per preparation
Few . . . . 4–10 per preparation
Moderate number . . . . 11–20 per preparation
Many . . . . 21–40 per preparation
Very many . . . . over 40 per preparation

Note: Plate 5.1 shows the relative sizes of helminth eggs and a *Strongyloides* larva in a saline preparation as seen in a microscope field viewed with the10_ objective and 10_ widefield eyepiece. Plate 5.2 shows the relative sizes of protozoan trophozoites, cysts, and oocysts in saline and iodine when viewed with the 40_ objective and 10_ eyepiece.

Identification of larvae: In a fresh faecal specimen, *S. stercoralis* is the only larva that will be found. It can be easily detected in a saline preparation by its motility and large size. If the specimen is not fresh, *S. stercoralis* will require differentiation from hookworm larvae (see subunit

Identification of helminth eggs: Eggs are recognized by their:

- size,
- colour (colourless, pale yellow, brown),
- morphological features.

The relative sizes and colour of the eggs of the common nematodes, flukes, and tapeworms are shown in Plate 5.2.

Blood Haemoglobin level measurement using HemoCue Hb 301

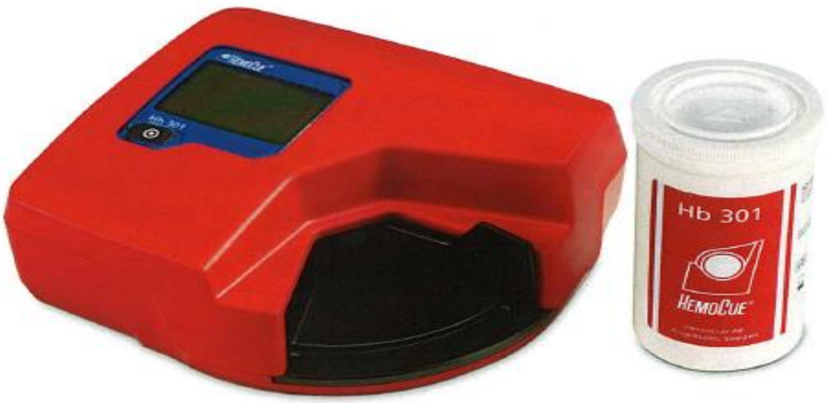

The Hb 301 system for point-of-care hemoglobin measurements is a swift and effective tool for anemia testing. Based on HemoCue's original cuvette technology, the system is optimized for use in primary care and blood donation settings.

- Cost-effective – clinically accurate results at economical conditions
- Fast – results in approximately 10 seconds
- Accurate and precise – no calibration or instrument adjustment needed
- Robust – designed for high temperatures and humidity
- Hygienic – single-use cuvettes and minimal exposure to blood
- User-friendly – easy to use with little training or supervision
- Portable – battery operated

Three simple steps

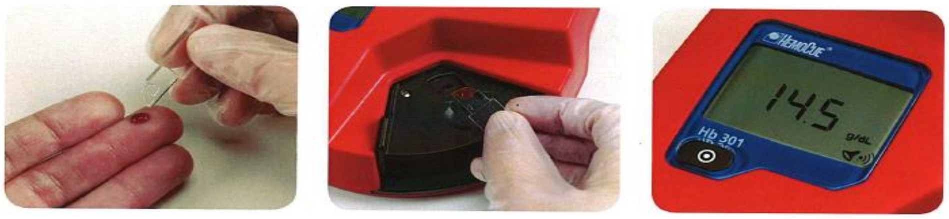

Fill the cuvette with a drop of blood, place it in the analyzer and receive a lab-quality result – in approximately 10 seconds.

HEMOCUE®
A Quest Diagnostics Company

Source: Setema Limited PLC and Monica Cheesbrough, 2nd edition, 2009
